# Effectiveness of functional orthodontic appliances in obstructive sleep apnea treatment in children: literature review

**DOI:** 10.1016/j.bjorl.2021.02.010

**Published:** 2021-03-14

**Authors:** Rita Catia Brás Bariani, Renato Bigliazzi, Mario Cappellette Junior, Gustavo Moreira, Reginaldo Raimundo Fujita

**Affiliations:** a Universidade Federal de São Paulo (Unifesp), Departamento de Otorrinolaringologia e Cirurgia de Cabeça e Pescoço, São Paulo, SP, Brazil; b Private Pratice in Orthodontics, São Paulo, SP, Brazil; c Universidade Federal de São Paulo (Unifesp), Departamento de Psicobiologia, São Paulo, SP, Brazil

**Keywords:** Obstructive sleep apnea, Upper airway resistance, Functional orthodontic appliance, Craniofacial abnormalities, Children

## Abstract

**Introduction:**

Obstructive sleep apnea syndrome is a common condition in childhood and if left untreated can result in many health problems. An accurate diagnosis of the etiology is crucial for obstructive sleep apnea treatment success. Functional orthodontic appliances that stimulate mandibular growth by forward mandibular positioning are an alternative therapeutic option in growing patients.

**Objective:**

To perform a literature review about the effects of functional orthodontic appliances used to correct the mandibular deficiency in obstructive sleep apnea treatment.

**Methods:**

The literature search was conducted in June 2020 using Cochrane Library; PubMed, EBSCO (Dentistry & Oral Sciences Source), LILACS Ovid; SciELO Web of Science; EMBASE Bireme and BBO Bireme electronic databases. The search included papers published in English, until June 2020, whose methodology referred to the types and effects of functional orthopedic appliances on obstructive sleep apnea treatment in children.

**Results:**

The search strategy identified thirteen articles; only four articles were randomized clinical studies. All studies using the oral appliances or functional orthopedic appliances for obstructive sleep apnea in children resulted in improvements in the apnea-hypopnea index score. The cephalometric (2D) and tomographic (3D) evaluations revealed enlargement of the upper airway and increase in the upper airspace, improving the respiratory function in the short term.

**Conclusion:**

Functional appliances may be an alternative treatment for obstructive sleep apnea, but it cannot be concluded that they are effective in treating pediatric obstructive sleep apnea. There are significant deficiencies in the existing evidence, mainly due to absence of control groups, small sample sizes, lack of randomization and no long-term results.

## Introduction

Obstructive sleep apnea syndrome (OSAS) in childhood is characterized by intermittent partial (obstructive hypopnea) or complete collapse of the upper airway (apnea) during sleep.^1^ OSAS is a common condition in childhood (ranging from 1.2% to 5.7%)^2^ and if left untreated can result in many health consequences including lethargy, memory loss, problems with thinking and judgment, disruption of normal metabolic functions, and cardiovascular disorders.^3^ Obstructive sleep apnea (OSA) in children differs in relation to adults regarding the pathophysiology, clinical picture, diagnosis and treatment.^4^ Pharyngeal and palatine tonsillar hypertrophy and obesity are the most common causes of the syndrome in childhood, but the complexity of OSAS is exemplified by other related factors involving the craniofacial structures and neuromuscular tone.^4^ OSA severity is heterogeneous among patients and the wide range of presentation leads to variations in management approach and differences in treatment response.^5^

The treatment of OSA is based on the child’s age, severity of symptoms, clinical findings, presence of comorbidities, and polysomnographic (PSG) findings.^6^ High clinical therapeutic effectiveness for OSA has been reported after adenotonsillectomy in nonobese children, and there is evidence of improvements in oximetry as well.^7^ Evidence-based guidelines support the use of continuous positive airway pressure treatment (CPAP) as an effective first-line treatment of OSA in children without adenotonsillar hypertrophy; however, this is complicated by low tolerance or high refusal level of treatment (25%–50%).^8^, ^9^, ^10^

Children with OSA with concomitant craniofacial risk factors should be referred to an orthodontist involved in a multidisciplinary sleep medicine team. Orthodontic treatment for correction of maxillomandibular anomalies or mandibular retrusion has been shown to improve OSA.^11^ Functional orthodontic appliances (FOA) are used for craniofacial abnormalities and may induce significant change in mandibular shape that leads to correction of dentoskeletal disharmony associated with mandibular retrusion.^12^ The nature of the variations that induce mandibular growth with functional appliances is not yet clear but orthopedic correction of mandibular retrognathism seems to increase the airway space in the short term in 3-dimensional (3D) perspective.^13^ Several studies in the literature have investigated the mechanisms of action and the effects of functional appliances and there is no evidence of contra-indications or even significant side effects as its use is short-term in nature.^14^ Recent systematic reviews and meta-analyses have shown that, in the short term, FOA produces greater skeletal mandibular effects when performed at puberty.^12^ In patients treated before the pubertal period, the significant effects seems to be confined to the dentoalveolar level, with minimal clinical implications.^15^

There are few studies evaluating the use of FOA and their effectiveness in children during sleep for OSAS.^16^ The aim of this study, therefore, was to perform a literature review about the effects of FOA used to correct the mandibular deficiency in OSA treatment.

## Methods

### Search strategy

Two authors (R.C.B.B. and R.B.) screened studies and extracted data independently in Cochrane Library; PubMed, EBSCO (Dentistry & Oral Sciences Source), LILACS Ovid; SciELO Web of Science; EMBASE Bireme and BBO Bireme electronic databases.

The following search strategy was used: apnea syndrome, sleep OR apnea syndromes, sleep OR apnea, sleep OR apneas, sleep OR breathing, sleep-disordered OR hypersomnia with periodic respiration OR hypopnea, sleep OR hypopneas, sleep OR mixed central and obstructive sleep apnea OR mixed sleep apnea OR mixed sleep apneas OR sleep apnea OR sleep apnea syndrome OR sleep apnea, mixed OR sleep apnea, mixed central and obstructive OR sleep apneas OR sleep apneas, mixed OR sleep disordered breathing OR sleep hypopnea OR sleep hypopneas OR sleep-disordered breathing OR sleep apnea OR sleep apnea OR sleep apnea syndrome OR sleep apnea syndrome OR snoring OR upper airway resistance syndrome AND intraoral OR intra-oral OR oral OR klammt OR bimler OR “functional orthodontic appliance” OR “functional orthopedic appliance” OR “activator appliance” OR “mandibular advancement appliance” OR “oral appliance” OR “kinetor appliance” OR “planas appliance” OR “bimler appliance” OR “frankel appliance” OR “frankel function regulator” OR “functional regulator” OR “harvold activator” OR “andresen appliance” OR “bass appliance” OR bionator OR “bite block” OR “twin block” OR “herbst appliance” OR “herren activator” OR “woodside activator” OR “dental device” OR “intraoral device” OR “oral device” OR “anterior mandibular positioning device” OR “tongue device” OR “mandibular device” OR “mandibular advancement device” OR “dental appliance” OR “tongue appliance” OR “mandibular appliance” OR “intraoral appliance” OR “mandibular advancement splint” OR “mandibular prosth*” OR correct* OR prevent* OR intercept* AND orthodont* AND device* OR mobile OR equipment OR appliance* OR removable OR orthodont*.

All reviewed articles and cross-referenced studies were screened for relevant data. A manual review of reference lists of included studies and previously published systematic reviews and meta-analyses on OSA and intraoral appliances was also conducted. No language restrictions were applied. Any disagreement was solved by consensus. All reviewed articles and cross-referenced studies were screened for relevant data.

### Inclusion criteria

The inclusion criteria were formulated according to the population, intervention, comparison, outcome, study design (PICOS)^17^ principle:

Population — Children and adolescents (14 years old or younger) diagnosed with OSA without craniofacial syndromes.

Intervention — FOA.

Comparison — With or without a control group or pre-treatment and post-treatment.

Outcome — Primary outcome was the apnea-hypopnea index (AHI); secondary outcomes were (1) oxygen saturation level, (2) sleep quality (SQ), (3) improvement on sagittal relationship between the maxilla-mandible measured by cephalometric data; and (4) upper-airway space.

Study design — Case reports, pilot studies, randomized (RCTs) and nonrandomized controlled trials.

Studies considered for inclusion were published in any language. As one of the outcomes is the AHI, polysomnography was mandatory for inclusion of the chosen articles.

### Data items and collection

The following data items were independently extracted from each included study by two reviewers: author, year of publication, study design, subjects, age, interventions, wearing time, drop out, AHI before and after FOA (only effects would be pooled), and secondary outcomes.

## Results

### Summary of included studies

**Figure 1:**
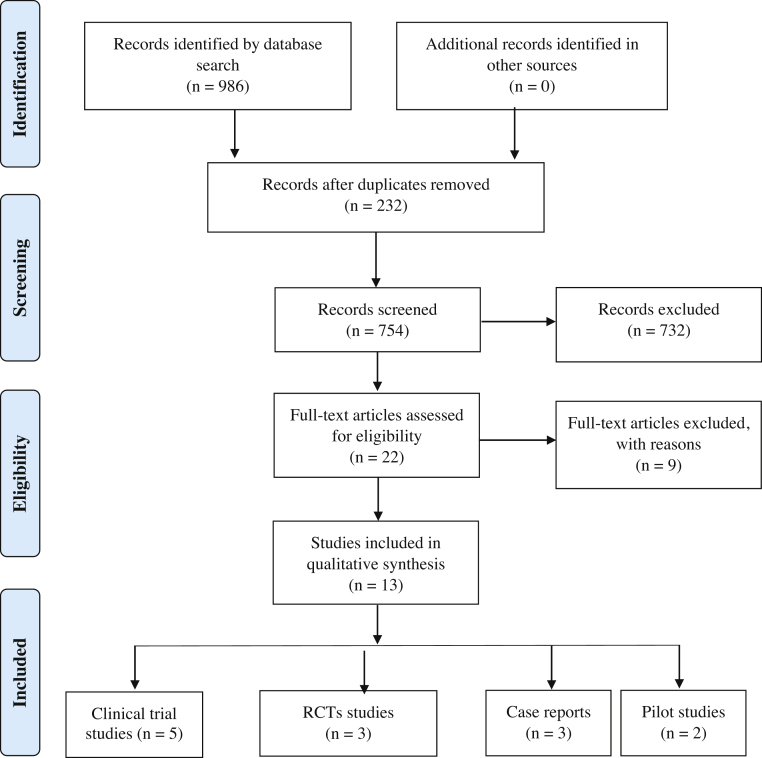
Flowchart of the selection process.

A flow diagram of the study identification, screening, eligibility, and inclusion is shown in Fig. 1. A total of 754 studies were identified and assessed for inclusion. After exclusion on the title and abstract stages, 22 articles were retrieved for full review. Nine were later excluded after full text review for different reasons. Therefore, only 13 articles met the inclusion criteria set for this study. Key methodological and descriptive characteristics of the included articles are presented in Table 1, Table 2, Table 3, Table 4.

**Table 1:** Distribution according to the type of study — clinical trials.

Author, year	Sample	Age	Appliance	Mandibular advancement	Treatment time	Evaluation	Results
Cozza et al.,^18^ 2004	20 patients: 10 boys, 10 girls	5.91 ± 1.14 years	Modified monoblock	The MM was produced from a construction bite that positioned the mandible anteriorly into an edge-to-edge incisal relationship. The lower jaw was postured forward to increase intermaxillary space. As a general rule, the bite registration was taken 3 mm short of maximum.	6-months	Polysomnography	AHI from 7.88 to 3.66. Improved daytime sleepiness and sleep quality
						Cephalometry	
						Plaster models	
						Epworth questionnaire	
Levrini et al., ^19^ 2018	9 patients	4–8 years	Miobrace	No information	3-months	Polysomnography	The difference between AHIs computed a statistically significant decrease (*p* = 0.0425) in the occurrence of apneas and hypopneas in the studied subjects. The differences between SaO_2_ before and after treatment reported an improvement in the percentages of oxygen saturation, but it did not reach a statistically significant difference.
Maspero et al., ^20^ 2015	40 patients, 10 controls	40 patients: the age range of 9–14 years (17 females between 8–10 years, and 23 males between 10–14 years). Ten control group subjects (n = 10, 5 females between 9–10 years and 5 males between 11–14 years)	Andresen activator	2–3 mm	16-months	Polysomnography	Snoring was reduced in all the treated patients. The follow-up polysomnography confirmed improved breathing parameters in the treated group while in the control group no changes were observed. Correction of mandibular retrusion in patients with a class II malocclusion can increase the sagittal dimension on the posterior oropharyngeal airway.
						CBCT	
Cozza et al.,^21^ 2004	20 patients, 20 control	5.91 ± 1.14 years	Modifiedmonoblock	The MM was produced from a construction bite that positioned the mandible anteriorly into an edge-to-edge incisal relationship. The lower jaw was postured forward to increase intermaxillary space. As a general rule, the bite registration was taken 3 mm short of maximum.	6-months	Polysomnography	Mean ± SD of ESS in patients with OSA before treatment was 15.2 ± 4.9 and after therapy it was reduced to 7.1 ± 2.0. The median apnea-hypopnea index was reduced after 6-months of therapy with the intraoral appliance, from 7.88 to 3.66 in all patients.
						Epworth questionnaire	
Zhang et al.,^22^ 2013	31 boys, 15 girls	9.7 ± 1.5 years	Twin-block	No information	10.8-months	Polysomnography	The mean AHI index decreased from 14.08 ± 4.25 to 3.39 ± 1.86 (*p* < 0.01), and the lowest SaO2 increased from 77.78 ± 3.38 to 93.63 ± 2, 66 (*p* < 0.01). Cephalometric measurements showed a significant increase in the upper posterior air space, medium air space, SNB angle and facial convexity, which indicate increased mandibular growth.
						Cephalometry	

AHI, apnea hypopnea index; ANB, A point-nasion-B point angle; ESS, Epworth sleepiness scale; OSA, obstructive sleep apnea; OSAS, obstructive sleep apnea syndrome; PSQ, pediatric sleep questionnaire; SaO_2_, arterial oxygen saturation; SD, standard deviation; SNB, sella-nasion-B point angle; SRBD, sleep-related breathing disorder; MM, modified monoblock.

**Table 2:** Distribution according to the type of study — randomized clinical trial (RCT).

Author, year	Sample	Age	Appliance	Mandibular advancement	Treatment time	Evaluation	Results
Villa et al.,^23^ 2002	14 patients, 9 controls	6.86 ± 2.34 years	Acrylic resin bite plate for mandibular positioning	5 mm	6-months	Polysomnography	Apnea index (*p* = 0.001) and hypopnea index (*p* = 0.001) decreased significantly. The clinical evaluation before and after treatment showed that, in 7 of the 14 individuals (50%) the oral appliance reduced the symptoms (drop of at least 2 respiratory points) and in 7 it had resolved the main respiratory symptoms. Effective and well tolerated.
						Modified Brouillette questionnaire	
Idris et al.,^24^ 2018	16 patients	9.8 ± 1.1 years	Twin-Block (MAS Active) Two removable upper and lower acrylic plates. (Sham MAS)	3 mm	3 weeks	PSQ	Compared to the Sham MAS, the wearing of the Active MAS resulted in a significant reduction in overall AHI (−37%; 95% CI 15%–53%; *p* = 0.002) and supine AHI (−4.1 events per hour; 95% CI 1.8–6.4; *p* < 0.001). Mean snoring time per night was shorter with the Active MAS than with the Sham MAS (−46.3 min; 95% CI 14.5–78.1; *p* = 0.004). Wearing of the Active MAS improved the ratings of quality of life and behavior (*p* ≤ 0.028), but there was no evidence that it influenced IGF-1 levels (*p* = 0.172).
					2 week washout period	OSA 18	
						BESS	
						ESS	
						Polysomnography	
						Serum levels of IGF-1	
Nunes Jr et al.,^25^ 2019	24 patients, 16 controls	6–9 years	BioAJustax oral appliance	Installation, a bite guide was molded with acrylic behind the upper incisors to place the lower incisors in a more anterior contact with the upper incisors favoring the advancement of the mandible to class I constructive bite.	6-months	The PSQ	Six months of OOA treatment in snoring children with SDB promotes the enlargement of pharyngeal dimensions and beneficial cephalometric changes. Snoring <0.001
						Nasofibroscopy	
						Polysomnography	
						Cephalometry	
							Mouth breathing <0.001

BESS, behavioral and emotional screening system; ESS, Epworth sleepiness scale; IGF-1, insulin-like growth factor-1; OOA, oral orthopedic appliance; OSA, obstructive sleep apnea; PSQ, pediatric sleep questionnaire; SDB, sleep disordered breathing; SRBD, sleep-related breathing disorder.

**Table 3:** Distribution according to the type of study — case report.

Author, year	Sample	Age	Appliance	Mandibular advancement	Treatment time	Evaluation	Results
Rădescu et al.^26^ 2017	1 girl	8-years	Twin block	The construction bite was recorded with a vertical opening of 2–3 mm between upper and lower incisor and with sagittal advancing of the mandible at an edge-to-edge incisor relationship	12-months	Polysomnography	The AHI increased significantly from 2.6 to 10.2 events per hour of sleep. The initial pre-treatment rate for apnea-hypopnea was 34 events/h of sleep and increased to 81 events/h in the sleep period, when the polymer appliances were used. Snoring intensity indexes increased from 4.2 to 51.3 events/h and the oxygen desaturation index has significantly variable changes from 3.2 to 10.8 events/h.
						Cephalometry	
Rose et al.,^27^ 2006	1 girl	1 girl with 8 years	Frankel II	Construction bite with a mandibular protrusion of a ½-width of a premolar and a bite opening of approximately 5 mm	Girl: 20 months	Polysomnography	Improvement in cardiorespiratory performance parameters, with less apnea and oxygen desaturation. While the OSAS in the first case could be eliminated long-term by the orthodontic procedure, it remains unclear in the second case whether the improved respiratory situation during sleep that will be maintained in the long run.
	1 boy	1 boy with 6,5 years			Boy: 9 months		
Schessl et al.,^28^ 2006	1 boy	3.5-year	Frankel II	5 mm	14-months	Polysomnography	Frankel II proved to be effective in the treatment of obstructive sleep breathing disorders in this specific clinical situation in children with primary dentition and without alteration of mandibular position. Oxycardiorespirography (Poly-MESAM) with videotaping revealed a flow pattern similar to repetitive obstructive apnea and an oxygen saturation periodically dropping to 80% for more than 70% of the entire sleep recording. After: While the device was being worn during the night, snoring, apneic episodes and daytime tiredness disappeared and the boy stopped napping in the morning. Oxycardiorespirography (PolyMESAM) 3 months later showed regular breathing for 80% of the night.

AHI, apnea hypopnea index.

**Table 4:** Distribution according to the type of study — pilot study.

Author, year	Sample	Age	Appliance	Mandibular advancement	Treatment time	Evaluation	Results
Modesti-Vedolin et al.,^29^ 2018	18 patients	8.3 ± 2.3 years	Thermoplastic intra oral device superior and inferior.	5–7 mm	2-months	Sleep Disorder Scale for Children DSC)	The average RDI was significantly reduced from 10 to 4.5 events/h. The Nadir SpO_2_ increased significantly from 82.6% to 88.9%. Total snoring events/hour also decreased.
						BiteStrip® the type 3 Portable device (ApneaLinkTM Plus)	
						Research Diagnostic Criteria for Temporomandibular Disorders	
Machado Junior et al.,^30^ 2016	8 patients, 6 controls	Patients 8.39 years controls 8.13 years	Planas Appliance Modified	No information	3-months	Polysomnography	There was a decrease in AHI one year after implementing the use of mandibular advancement devices, in comparison with the group that did not use these devices. AHI1 (before) 1.66, AHI2 (after) 0.30

RDI, respiratory disturbance index; AHI, apnea hypopnea index; SaO_2_, arterial oxygen saturation; SRBD, sleep-related breathing disorder.

All the included articles were published between 2002 and 2019 and were in English language except for one article in German.^28^ The study included 13 articles, and a summary of study characteristics and results of the studies is shown in Tables: five clinical trial studies (Table 1), three RCTs (Table 2), three case reports (Table 3) and two pilot studies (Table 4).

All included studies investigated 271 growing subjects (range 3.5–14 years), with mean age of 7.61 ± 1.99 years. As with age, the mean treatment observation varied widely between studies (range 1–20 months), with treatment time of 7.71 ± 5.13 months on average. As for the type of removable appliances, the most used was the Twin Block,^22^, ^24^, ^26^ Frankel II,^27^, ^28^ and Modified Monoblock.^18^, ^21^ Three studies^19^, ^22^, ^30^ did not report the amount of mandibular advancement during treatment, while in three others,^18^, ^21^, ^25^ a single mandibular advancement to an incisor end-to-end relationship was performed. In the other studies included, mandibular advancement varied from 3 to 7 mm.

Regarding the AHI index changes, twelve studies reported reduced AHI after treatment, even though this conclusion could not be statistically reached due to the considerable heterogeneity of pooled data. Only Rădescu et al.,^26^ in a case report, found a negative correlation between AHI and FOA. Villa et al.,^23^ summarized sleep quality (SQ) data as daytime and nighttime symptoms, expressed as the percentage of positive reports among treated subjects. These administered questionnaires showed diminished symptoms following 6 months of treatment. Conversely, Cozza et al.,^18^, ^21^ discussed reduced daytime sleepiness following treatment, but without reporting any data. Overall, the appliances were well tolerated.

## Discussion

Effective treatment for OSA in children should be focused on one or more risk factors to help cure the obstruction. An accurate diagnosis of the etiology of OSA is crucial for the treatment success. Conditions such as obesity, adenoid hypertrophy, craniofacial abnormalities, and other factors could narrow the anatomic airway.^31^ A significant number of children with OSA do not respond favorably to the primary treatment “adenotonsillectomy” or do not tolerate CPAP treatment. Removable functional appliances are less invasive and can be better tolerated than other modalities.^24^ OSA has been associated with deviations in craniofacial growth in children. Maxillary constriction and skeletal class II with retruded small mandible and hyperdivergent pattern have been widely accepted as dominant risk factors of OSA.^32^, ^33^, ^34^ An et al.^35^ emphasize that the strength of the relationship between these craniofacial morphologies and the development of OSA is not well established. The authors^35^ identified three phenotypes in OSA adults based on clustering using craniofacial variables in relation to OSA severity and obesity and characterized the phenotypes by differential correlation factors to OSA severity (AHI): Cluster-1, obesity type, Cluster-2, skeletal type, and Cluster-3, complex type. The patients in Cluster-2, who have collapsible upper airway primarily driven by craniofacial anatomic vulnerability without non-anatomic problems, would be the best indication of orthopaedic or surgical modification of craniofacial structure.

FOA has been used for many decades to correct mandibular retrognathism. To treat some types of malocclusion, the mandible posture is previously changed to stimulate mandibular growth, especially in cases of retrognathism. Functional treatment stimulates mandibular growth by forward posturing of the mandible with the condyles displaced downward and forward in the glenoid fossa.^36^, ^37^ This change will also transform the relationship between all structures adjacent to the mandible, also increasing the dimensions of the upper airways. Growing adolescents with skeletal class II malocclusions treated with functional appliances demonstrated an increase in pharyngeal airway dimensions of the oropharyngeal region, and such changes were consistently maintained even after growth completion.^32^, ^38^

The aim of this review was to evaluate the types of FOA and their effectiveness for sleep apnea in children. Few prospective and randomized clinical studies with methodological quality have been identified and included in this study. Villa et al. in 2002^23^ reports that, in addition to treating the craniofacial problem, FOA would also be treating OSA because they promote mandibular replacement during sleep and increase the retroglossal space by anterior displacement of the tongue, improving respiratory function, especially at night. Therefore, early treatment of craniofacial abnormalities can prevent the development of long-term respiratory failure, impacting the quality of life in adulthood.^23^, ^34^, ^39^, ^40^, ^41^

Several randomized clinical trials^23^, ^24^, ^25^ suggest that orthodontic treatments, such as mandibular advancement with functional appliances, can be effective in the management of pediatric snoring and OSA. Thus, these results indicate that correcting craniofacial structure imbalances during growth can reduce snoring and OSA in children and adolescents. Thus, orthodontic treatment using FOA is considered a potential additional treatment for pediatric OSA for all included studies.

The amount of mandibular advancement in FOA construction varies across patients and this is evident in the great variation reported in the studies of our review. In case of limited overjet, the bite can be registered by placing incisors in an edge-to-edge relation, while in case of large overjet the bite is usually registered 2–3 times by advancing the mandible gradually (step by step) in a limited of 4 mm per jump, which brings greater orthopedic changes ^37^ and obviously has a positive impact on the improvement of oropharyngeal conditions. The studies included in this review used different appliances to achieve mandibular advancement but similar results according to the type of appliance it was observed. Within the limitations and heterogeneity of the included studies it appears that, despite the specific type of appliance used and the protocol followed, we observed a reduced AHI index after treatment, with reports of improving daytime sleepiness and sleep quality, decreasing snoring and the mouth breathing and promotion of the enlargement of pharyngeal dimensions and beneficial cephalometric changes. No study can be included in our review to discuss the impact of FOA treatment in long-term observation period.

Removable functional appliances can help improve the permeability of the upper airway during sleep, widening and decreasing the collapse of the upper airway, thus increasing its muscle tone.^32^ FOA therapy should be encouraged in pediatric OSA, and an early approach can permanently change breathing and nasal breathing, thereby preventing upper airway obstruction.^42^

Our literature review found low-quality evidence to support the use of mandibular advancement appliances in managing obstructive sleep apnea in children.^43^ The different therapeutic effects of a FOA in the treatment of obstructive sleep disorders might be due to differences in study protocols, appliance design and subject selection.

The orthodontist should be part of the health professional team involved in the multidisciplinary treatment of OSAS because, when treating malocclusion and craniofacial orthopedic problems, they may eventually be treating the respiratory problems of their patients.

## Conclusion

FJO can be considered as a potential additional treatment in children with OSA, but more randomized studies are necessary with larger sample sizes involving a representative number of patients with apnea and malocclusion to establish protocols related to the time of use of the appliance per day, total treatment duration and long-term comparison of the effects of different types of FOA.

## Funding

Associação de Incentivo à Pesquisa (10.13039/501100006303AFIP), Coordenação de Aperfeiçoamento de Pessoal de Nível Superior (10.13039/501100002322CAPES) - provided material and financial support. Number 88882.430440/2019-01.

## Conflicts of interest

The authors declare no conflicts of interest.
